# Development and Psychometric Evaluation of a Musical Profile Questionnaire (MPQ): A Contribution for Music Education

**DOI:** 10.3390/bs15070901

**Published:** 2025-07-03

**Authors:** José Salvador Blasco-Magraner, Gemma Ruiz-Varela, Pablo Marín-Liébana, Gloria Bernabe-Valero

**Affiliations:** 1 Facultad de Magisterio, Universidad de Valencia, 46010 Valencia, Spain; j.salvador.blasco@uv.es (J.S.B.-M.); pablo.marin-liebana@uv.es (P.M.-L.); 2 Facultad de Magisterio, Universidad Alfonso X El Sabio, 28691 Madrid, Spain; gemmaruiz@uax.es; 3 Facultad de Psicología, Universidad Católica de Valencia “San Vicente Mártir”, 46001 Valencia, Spain

**Keywords:** music psychology, music education, psychometric properties, musical self-efficacy, musical identity

## Abstract

The development of a Spanish musical profile questionnaire, designed to assess both objective aspects of musical engagement and psychological dimensions, represents a significant advancement in the fields of music psychology and music education. Grounded in prior research and created by experts in music education, this instrument offers a comprehensive portrait of musicians by capturing their training, experience, and psychological characteristics. A total of 261 musicians, ranging in age from 16 to 61 years, were recruited through incidental sampling to complete the questionnaire. The final version encompasses both objective indicators of musical dedication and a psychological profile scale. The psychometric evaluation of the scale supported a robust three-factor model comprising “Musical Self-Perception”, “Personal Cost of Music”, and “Existential Contribution of Music”. The 10-item scale demonstrated acceptable internal consistency, along with strong criterion and convergent validity, affirming the instrument’s reliability. The discussion regarding the inclusion or exclusion of items related to theoretical content reflects an evidence-based approach aimed at continuous refinement of the tool. Furthermore, the identification of future directions, including exploration of additional psychological dimensions and potential adaptations for diverse cultural or educational contexts, underscores a forward-looking commitment to the ongoing development and applicability of the questionnaire.

## 1. Introduction

Music can play a significant role in the formation of individual identity, in some cases with an intensity and depth that surpass other types of personal experiences or everyday activities (Bowman, 2004; Gruhn et al., 2017). Within this framework, musical identity is understood as a psychological construct that reflects how individuals perceive themselves in relation to musical practices, roles, and experiences. It integrates personal, social, emotional, and musical components, contributing to the development of what Gruhn et al. (2017) describe as the musical self, a dynamic and multifaceted self-concept shaped by continuous interaction with musical environments.

Psychology of music is the scientific study of how music affects human thoughts, emotions, and behavior. Based on this, music psychology offers both theoretical and empirical insights into how engaging with music contributes to the formation and development of personal identity. This field explores how individuals’ musical behaviors, experiences, motivations, and emotions contribute to their psychological development and sense of self (Hallam, 2010; MacDonald et al., 2002). Nevertheless, it is important to note that the present study adopts as its main theoretical framework the models developed by MacDonald et al. (2002), which consider musical identity to be a dynamic construction that emerges from the interaction between personal, social, and musical factors. From this perspective, music not only influences the development of the self, but also serves as a means of exploring and consolidating identity. In this way, musicians gradually incorporate musical experiences that shape their personal characteristics as musicians.

There is a high degree of overlap among terms related to musicians’ characteristics, often referred to as musical identity. This is acknowledged by Burland et al. (2022), who state that the descriptions and definitions of musical identity become quite diffuse and elusive, influenced by the semantic history of the term “identity” more generally (Gleason, 1983). Although it is difficult to find precise definitions for the various terms related to musicians’ characteristics, we provide several definitions of these concepts for clarification:

(1) Musical identity. MacDonald et al. (2002) approach musical identity from a psychological perspective, exploring how music influences the construction of personal and social identity. In summary, according to MacDonald et al. (2002), musical identity refers to the way in which music contributes to the formation and expression of both individual and collective identity, serving as a means of communicating and negotiating fundamental aspects of the self.

(2) Musical self-concept. Vispoel (1995) defines musical self-concept as a specific facet of artistic self-concept, which is itself integrated into a hierarchical and multidimensional model of self-concept. In his proposal, he extends the hierarchical model of Shavelson et al. (1976) to the artistic domain, suggesting that musical self-concept not only involves an evaluation of specific musical skills, such as singing or instrumental performance, but also forms part of a broader system of artistic self-perceptions that vary depending on individual experiences and are hierarchically organized from specific aspects to a general artistic self-concept.

For their part, Spychiger et al. (2009) propose an even broader model of musical self-concept that incorporates both academic dimensions (what one can do) and non-academic ones (who I am in relation to music). Their approach highlights emotional, social, cognitive, and spiritual aspects, emphasizing that musical self-concept is not limited to technical skills, but also reflects musical identity and one’s personal connection with music.

Therefore, musical self-concept is the personal and multidimensional perception an individual has of their musical abilities, identity, and relationship with music, and it plays a key role in their musical development and participation. Musical self-concept integrates perceptions, beliefs, and self-schemas about a person’s musical abilities and potential (Schnare et al., 2011; Vispoel, 1995).

(3) Musical self-perception. Morin et al. (2016) define musical self-perception as an integration of perceptions, beliefs, and self-schemas about a person’s musical abilities and potential. This construct is characterized as multifaceted, hierarchically organized, and influential in one’s motivation toward musical practice. These authors developed and validated a short version of the Music Self-Perception Inventory (MUSPI), which assesses six specific dimensions of musical self-concept: composing, listening, dancing, playing instruments, reading music, and singing. In addition, a subscale is included to assess the overall perception of musical self-concept. In summary, musical self-perception refers to the way a person perceives and evaluates their musical skills across various specific areas, and how these perceptions influence their motivation and participation in musical activities.

(4) Musical dedication. Musical dedication refers to the level of commitment, sustained effort, and priority that a person assigns to the practice, study, and participation in musical activities, whether in a professional, academic, or recreational context. This construct involves motivational, affective, and behavioral components that reflect the importance of music in the individual’s life and their willingness to invest time and resources in their musical development (Sloboda et al., 1996).

According to Hargreaves et al. (2003), musical identities are not singular or static; rather, they represent the ways individuals locate themselves within the social and cultural roles present in the musical domain, such as performer, student, teacher, or composer, and are influenced by broader educational and professional trajectories. Similarly, Dys et al. (2017) emphasize that musical identity also affects preferences and future engagement with specific styles or genres of music.

Musical identity, therefore, is dynamic and multifaceted. Individuals can simultaneously maintain multiple musical identities, even within a single professional category. As Hennion (2011) points out, a person may identify concurrently as a performer, composer, and music educator, depending on their social and professional contexts. Within the musical field, there are various roles, such as conductor, teacher, critic, or musicologist, among others. Kemp (1996) supports this perspective by demonstrating that musicians from different instrumental families—strings, brass, percussion, woodwinds—exhibit unique personality profiles, highlighting how the nature of musical engagement shapes identity in nuanced and specific ways.

Music students develop musical skills that are essential for their future professional careers and for obtaining symbolic recognition, which in turn contributes to the formation of their professional self-concept (Kadushin, 1969; O’Neill, 2002). Moreover, musical identity is closely tied to other aspects of self-image, interacting with dimensions such as gender (Dibben, 2002; Rolvsjord & Halstead, 2013), age (Whiteley, 2003), national identity (Folkestad, 2002; Hudson, 2006), disability (Howe et al., 2016), and personal identity more broadly (Green, 2011; Roberts, 1991). These intersections underscore the relevance of music psychology in understanding how individuals locate themselves musically within wider social and psychological frameworks.

Despite its importance, there are currently few instruments that comprehensively measure musicians’ profiles. In this regard, Gruhn et al. (2017) developed the Music Identity Scale (MIS), an instrument that explores social, personal, musical, and educational implications of identity, particularly among music educators and performers. Burland et al. (2022) developed the Musical Identity Measure (MIM), which incorporates motivational elements as central components of its framework. These elements are reflected in four factors: musical calling, musical self-efficacy, emotional attachment, and growth mindset. Musical calling captures a profound sense of purpose and intrinsic motivation to engage with music as a core aspect of one’s identity. Musical self-efficacy refers to the belief in one’s musical abilities, which fosters sustained effort and confidence in performing musical tasks. Emotional attachment represents the affective bond with music, reinforcing engagement through emotional fulfillment and personal meaning. Finally, growth mindset reflects the belief that musical abilities can be developed through dedication and perseverance, promoting resilience and long-term commitment. Collectively, these components demonstrate that motivation is not a peripheral aspect but a fundamental element in the conceptualization of musical identity within this model.

There are also instruments to measure more specific aspects, for example, musical self-efficacy (Ekinci, 2013; Ritchie & Williamon, 2007; Zelenak, 2010) and musical self-concept (Granda Vera et al., 2017; Hash, 2017; Morin et al., 2016; Spychiger et al., 2009), which can be useful for exploring the various relationships between related musical concepts.

However, none of the aforementioned scales assess both the structural (behavioral–experiential) and psychological (cognitive–emotional) dimensions of musical identity within a single questionnaire, allowing for a more comprehensive characterization of musicians. This refers to the evaluation of how much of a musician a person is and what their musical experience is like, aspects that can be analyzed through objective indicators such as the time spent on musical practice and the variety of areas in which they are involved, as well as through a psychological musical profile that considers cognitive–emotional aspects related to the musician’s identity.

In this regard, we consider it useful to develop the Musical Profile Questionnaire (MPQ), which aims to describe musicians based on two main dimensions: an objective or structural dimension, encompassing aspects such as time dedicated to and the scope of musical activity, referred to as the Structural Musical Profile (SMP); and a subjective dimension, referred to as the Psychological Musical Profile (PMP). Specifically, the PMP is a psychometric scale that encompasses a set of cognitive–emotional elements reflecting an individual’s subjective relationship with music, including their musical self-perception, motivational factors, and the personal significance they attribute to music in their life. This concept differs from some musical identity questionnaires in that it focuses specifically on the emotional and motivational aspects of connection with music, beyond social roles or musical competencies. The integration of these two dimensions allows for the construction of a comprehensive profile for each musician, facilitating various forms of classification and comparison, and enabling a more precise categorization of this population.

It is worth mentioning the usefulness of an instrument with these characteristics, as the scores obtained will allow for the characterization of musicians and, consequently, the association of these profiles with multiple psychosocial variables. In this way, an instrument has been developed targeting musicians, defined as students of formal music education at conservatories, professional music educators, professional music performers, and amateur music performers. In this regard, the present study aims to present the process of constructing a questionnaire and analyzing the psychometric properties of an instrument designed to characterize the musical profile of participants, including both objective elements related to musical dedication and the psychological factors involved.

## 2. Materials and Methods

### 2.1. Participants

A total of 261 Spanish musicians participated, including 165 women and 96 men, with ages ranging from 16 to 61 years (M = 27.04, SD = 11.52). Participants were recruited through non-random incidental sampling. To ensure their status as musicians, they were asked whether they had formal music education and whether they considered music an important part of their identity. Affirmative responses to both questions were the inclusion criteria for the study. Regarding formal music education from a state-regulated conservatory, 38.9% had completed the elementary level, 43.8% the intermediate level, and 14.6% the advanced level. The remaining 2.6% had no formal music education but were self-taught or had studied at non-official academies. Participants received the research form via an online survey link, where they were informed about the research objectives, the anonymous and voluntary nature of their participation, and were asked for their informed consent to participate, in accordance with the ethical principles of the Helsinki Declaration (World Medical Association, 2015).

### 2.2. Materials

The Musical Profile Questionnaire (MPQ) was used, which is the main object of study in this work, and whose theoretical and psychometric properties are detailed in the procedure and results sections. This questionnaire consists of a structural component, with questions about objective elements (Structural Musical Profile dimension, SMP), and a psychological component, measured using a scale (Psychological Musical Profile Scale, PMP).

For convergent validity with the PMP scale, the General Musical Self-efficacy Scale by Ritchie and Williamon (2007, 2011), adapted to the musical context in Spanish by Cuartero-Oliveros (2019), was used. It consists of 22 items, subdivided into two scales of 11 items each: Self-efficacy for learning (e.g., item: “I am confident that I can prepare the repertoire well for this performance”) and Self-efficacy for performance (e.g., item: “I have set high goals for this performance, but I will likely not be able to achieve them”). The items are answered on a Likert scale with 7 response levels (1 Strongly disagree–7 Strongly agree). The internal consistency for AA in its adaptation is good (Cronbach’s α = 0.820) and acceptable for AIM (Cronbach’s α = 0.780).

### 2.3. Procedure

The construction of the MPQ followed the guidelines of Muñiz and Fonseca-Pedrero (2019), which in turn incorporate the recommendations from the latest standards of the American Educational Research Association (AERA), the American Psychological Association (APA), and the National Council on Measurement in Education (NCME) (American Educational Research Association et al., 2014). In addition, approval was sought from an Ethics Committee for research, obtaining the ethical code UCV2017-18-28.

#### 2.3.1. Definition of the Measured Variables

To develop the assessment instrument, a review of the previous literature was conducted regarding the constructs that allow for a musical characterization in its psychological and structural spheres. This review was carried out by two expert musicians in music education, who later became part of the team responsible for creating the items. This item creation process led to the development of a general questionnaire composed of three instruments: (1) descriptive questions about the musicians; (2) questions regarding the objective elements of the musical profile, Structural Musical Profile (SMP); (3) questions about the psychological musical profile, related to various motivational aspects of musical identity, Psychological Musical Profile (PMP).

To gather information on the objective elements of the musical profile, items were created related to the achievement of formal academic music accomplishments and the personal history of hours dedicated to the study and practice of music, collecting information from childhood to the present, across various musical activity domains. Thus, when referring to the Structural Musical Profile (SMP), we are characterizing the time (both current and historical) and the domain of dedication of the musician.

To explore the psychological elements, items were developed to assess different aspects of musical self-efficacy, musical identity, and personal beliefs about music. These constructs have been highly relevant in the field of music education and music psychology (Cuartero-Oliveros, 2019; Reynolds, 1993; Sanders, 1998) and are fundamental for the characterization of the Psychological Musical Profile (PMP).

Thus, the construct of self-efficacy is defined as the belief in one’s ability to organize and execute the actions necessary to manage future situations (Bandura, 1977). This construct has been of great importance due to its influence on human behavior, motivation, and, consequently, achievement, and has been widely used to study the socio-cognitive processes involved in musical competence (Ritchie & Williamon, 2007, 2011; Sherer et al., 1982; Zelenak, 2010). This is because the constant effort required to develop musical ability, whose progress is often not immediate, demands high persistence (McPherson & McCormick, 2006). Items were developed based on the proposal by Rozalén (2009), who states that self-efficacy beliefs affect people’s productivity by regulating their behavior through four processes: cognitive (e.g., item: “I believe I have great musical ability”); motivational (e.g., “I put a lot of effort into music-related activities”); affective (e.g., “I am satisfied with the achievements I have made in relation to music”); and selection (e.g., “I believe that music has required a lot of sacrifice in my life”). Moreover, these items align with Bem’s (1972) Self-Perception Theory, which posits that individuals infer their traits and abilities from their actions and reactions. This implies that the perception of one’s own musical ability can be based on the observation of one’s dedication and achievements in musical activities. Similar to self-efficacy beliefs, musical self-perception is built and reinforced through personal experience and reflections on actions related to music, contributing to a more comprehensive and authentic view of one’s musical profile (Hargreaves et al., 2002; Sandgren, 2019). It is worth mentioning that, within the affective processes, an item from the field of positive psychology was also included, specifically regarding gratitude (“I am grateful for having had the opportunity to study music”), as it is considered an existential attitude with significant importance in the musical domain (Bernabe-Valero et al., 2019).

Regarding musical self-concept, two items were included (e.g., “Being a musician is part of my identity” and “The importance of music in my life is…”), which characterize the identity aspect of the individual’s musical dimension. Another fundamental aspect of the psychological dimension is the existential meaning of music: several authors have proposed that music impacts the lives of musicians, providing meaning, values, a sense of life, fulfillment, and life motivations (Bakan, 2019; Vernia, 2022). However, there is limited empirical research specifically exploring music as a source of meaning in life for musicians. Although there are studies on the effects of music on well-being, identity, cognition, and emotion, the direct exploration of the existential meaning of being a musician from an empirical perspective is scarcer. Therefore, it seemed relevant to include items to capture this content. These items were phrased in a counterfactual manner (e.g., “At times, I have thought that in a life without music, I would have enjoyed more freedom”) due to the advantages it provides. By presenting scenarios without music, the impact of music on the individual’s identity and well-being is indirectly highlighted (Epstude & Roese, 2008). This approach encourages deep reflection on the role of music in the person’s life, capturing a more implicit and authentic evaluation, and avoiding socially desirable responses that may occur in items that directly ask about the importance of music. Furthermore, this type of item allows for distinguishing between those who perceive music as essential and those who consider it dispensable, without the need for explicit questioning.

In summary, the PMP scale refers to the cognitive–emotional characterization of musicians. Appendix A includes the items and the dimensions to which they belong. Thus, it is expected that these dimensions will show relationships with each other, as greater time and breadth of dedication are expected to result in higher scores in the psychological musical profile. Additionally, it is expected that these dimensions will show relationships with each other and with the General Musical Self-efficacy Scale (Ritchie & Williamon, 2007, 2011; Cuartero-Oliveros, 2019), as this scale represents the socio-cognitive aspect included in the PMP scale.

#### 2.3.2. Specifications

This questionnaire has been developed for individual or collective application, primarily in an online format, although its application on paper is not excluded if necessary. The application form includes the necessary explanations for the correct understanding of the instructions, items, and issues related to the research and informed consent.

Care has been taken to ensure that the order of presentation of the questions is coherent and follows a logical flow to improve comprehensibility. Although there are some open-ended questions (e.g., activities related to music, instrument…), the majority are answered using a Likert-type scale, facilitating ease of response via mobile devices. The design prioritizes the brevity of the questionnaire, making it more versatile and useful for various research studies, in which it will likely be administered alongside other instruments. It is estimated that completing the questionnaire takes approximately 7 min.

The questionnaire has been developed for musicians, and the vocabulary used is understandable for anyone who has developed a level of musical competence similar to the elementary level of state-regulated conservatories. Regarding the age range, it is considered suitable for participants aged 12 years and older; however, for individuals under 18, there are structural dimension questions that will not be answered (e.g., those related to hours dedicated to music study from the age of 18 years). Researchers must account for this aspect and prepare the form based on the age group being studied in their research.

#### 2.3.3. Construction of the Items

A team consisting of three music experts in music education and one psychologist specialized in music psychology proceeded to develop an initial pool of 18 items for the structural dimension and 12 items for the psychological dimension, based on the dimensions previously outlined. Once the items were written, they were reviewed by four experts who provided feedback on (1) the clarity of item phrasing (comprehensibility for musicians as the target population), (2) the relevance of the items to the specified dimensions, and (3) whether the items were representative in characterizing the musical profile.

Six questions were designed to determine descriptive variables that provide relevant information about the musicians: instrument, level of education in music, age of onset, dropout from music studies, etc. For the structural dimension, items were created with a brief response format for time-related issues (e.g., identifying music study hours) and categorical responses for domains (YES/NO). For the psychological dimension, 10 questions were used to form the scale, with responses based on a 7-point Likert scale. These are the questions that have been analyzed to obtain the psychometric properties of the questionnaire.

#### 2.3.4. Edition

The online form presentation was reviewed to ensure proper formatting issues, the absence of typographical errors, and the inclusion of elements appealing to musicians (e.g., an image of a musician playing an instrument). A primary concern was ensuring that the form was clear and self-explanatory, as in some cases the instrument would be administered autonomously without the guidance of the researcher. To clarify any doubts, the email address of the principal investigator was provided for participants to contact if needed. Additionally, it was ensured that the responses from the form would be incorporated into an easily editable database for later analysis (a Microsoft Excel spreadsheet).

#### 2.3.5. Pilot Studies

A pilot study was conducted with 32 participants, students enrolled in a Bachelor’s Degree in Education, specializing in Music Education. This qualitative pilot study helped identify potential issues with item comprehension (e.g., in items 5–8, it was clarified that participants were asked for an estimate of time, not a sum, as some participants inquired about this). As a result, further explanation was added to some items to increase their clarity. The time taken to complete the questionnaire was also estimated, which was approximately 7 min. This pilot test confirmed that the questionnaire was clear and suitable to proceed with the main participant administration.

#### 2.3.6. Selection of Other Measurement Instruments

For convergent validity, the General Musical Self-efficacy Scale (Ritchie & Williamon, 2007, 2011; Cuartero-Oliveros, 2019), was selected, as it is conceptually close to the psychological musical profile dimension, representing musical self-efficacy. It was chosen because it is one of the few musical self-efficacy instruments found with a published Spanish version that indicates appropriate psychometric properties.

#### 2.3.7. Test Administration

Since the questionnaire was designed to be used with a broad range of musicians, sampling was carried out in various contexts, including university music students from different degree programs (e.g., psychology, education), conservatory students and teachers, musicians in musical bands, etc. Additionally, each musician was asked to share the form with other musicians from their context. The goal was to ensure the representation of the variety of musical profiles. Following the recommendations of Ferrando and Anguiano-Carrasco (2010), responses were collected from more than 200 participants.

### 2.4. Data Analysis

To describe the psychometric properties of the instrument, statistical analyses were conducted using JASP (version 0.16.1) and SPSS (version 28). Specifically, descriptive statistics (means, standard deviation, skewness, and kurtosis) and reliability analyses in terms of internal consistency were performed using Cronbach’s α and McDonald’s ω, as well as item-total correlations and factor analyses (Exploratory Factor Analysis and Confirmatory Factor Analysis for the single-factor model). For convergent validity analyses, correlations were conducted between the total PMP scale score and the subscales of the General Musical Self-efficacy Scale (Spearman’s Rho). For criterion-related validity, correlations (Spearman’s Rho) were performed between the PMP scale and structural dimension variables (music degree attained and areas of dedication in music).

## 3. Results

### 3.1. Psychometric Properties of the Psychological Musical Profile Scale (PMP)

Table 1 presents the descriptive statistics for the 10 items. The internal consistency was acceptable: Cronbach’s α = 0.782 and McDonald’s ω = 0.788. Both of these values were within the defined confidence intervals (α = 95% CI lower bound: 0.741; 95% CI upper bound: 0.817; ω = 95% CI lower bound: 0.749; 95% CI upper bound: 0.827). To assess homogeneity, the item-test correlations were analyzed. All 10 items from the initial scale demonstrated significant correlations with the total score (*p* < 0.01), as detailed in Table 1. Among these, six items exhibited very good homogeneity (scores > 0.4), three items showed moderate scores (>0.2 and <0.4), and one item had low scores (<0.2). These findings demonstrate that the 10 proposed items are significantly related to the total scale, indicating that they are measuring the same construct.

### 3.2. Exploratory Factor Analysis

The Shapiro–Wilk test yielded a *p* < 0.001 for all items, indicating non-normality of the data for all items, which led to the decision to apply the Principal Axis Factoring (PAF) method in the EFA with oblimin rotation, considering that the factors might be related. The KMO test for the 10-item EPM produced a satisfactory value of 0.78, and Bartlett’s test of sphericity was statistically significant, χ^2^(45) = 296.321, *p* < 0.001. The correlation matrix appears in Table 2. A single factor test was conducted using Confirmatory Factor Analysis to assess whether the instrument might be influenced by a single factor bias; the findings suggest that the instrument does not fit well with this structure (CFI = 0.64 and RMSEA = 0.18). In this way, the previous analyses indicate that our data yield good results for factorization, having confirmed that they are not subject to the possible bias of a single factor, and indicating the best method for EFA.

The exploratory factor analysis (EFA) conducted on the three-factor model revealed an explained variance of 53.6%. The goodness of fit was found to be significant, with χ^2^(18) = 48.51 and *p* < 0.001, leading to the acceptance of the model. These findings indicate that the EFA of this instrument yields a model with good psychometric properties, with a three-factor structure.

Table 3 presents the factor loadings, illustrating how each variable uniquely contributes to its corresponding factor. The oblique rotation of the factor solution indicated the existence of three latent factors that include all variables, with factor loadings exceeding the recommended inclusion criteria of 0.30 or 0.40 (Bandalos & Finney, 2018). These findings indicate that each item contributes to a unique factor, with all items exceeding the established inclusion criteria. Furthermore, it can be observed that the structure of the instrument is very clear, with no items contributing to more than one factor.

Reliability analyses were performed for the three factors. The first factor demonstrated good internal consistency (Cronbach’s α = 0.838 and McDonald’s ω = 0.847), while the second factor also showed good internal consistency (Cronbach’s α = 0.844). The third factor exhibited moderate consistency (Cronbach’s α = 0.680). These findings confirm that, with the three-factor structure proposed by the EFA, the reliability of the instrument remains valid.

In relation to criterion validity, the correlation score between the level achieved in music and the psychological profile dimension based on Spearman’s Rho was moderate (ρ = 0.43, *p* < 0.001). The correlation between the psychological profile dimension and the total number of areas of musical involvement was ρ = 0.56 and, with the current number of hours dedicated to music, it was ρ = 0.51, both significant at *p* < 0.001.

The correlation between the psychological profile dimension and the areas of dedication was 0.56 and, with the current hours of dedication to music, it was 0.51, both with a significance level of *p* < 0.001. In turn, the PMP scale correlated highly and significantly with the dimensions of the General Musical Self-efficacy Scale, both the subscale of Self-efficacy for Learning (ρ = 0.73) and the subscale for Self-efficacy for Performance (ρ = 0.97), with a *p*-value < 0.001, indicating good convergent validity.

These findings indicate that the PMP has good criterion validity by significantly correlating with the total number of areas of musical involvement and with the current number of hours dedicated to music, and good convergent validity by correlating with the self-efficacy scales.

### 3.3. Final Version of the Musical Profile Questionnaire (MPQ)

The final version of the questionnaire is presented in Appendix A. Below are the characteristics of the questionnaire. The MPQ aims to describe musicians primarily based on two dimensions: one objective or structural (including time and scope), which we referred to as the Structural Musical Profile (SMP), and one subjective, the Psychological Musical Profile (PMP). These dimensions together create the profile of each musician, allowing for numerous classifications and comparisons, potentially categorizing this population.

The SMP is a questionnaire with brief and dichotomous questions. The PMP is a psychometric scale, composed of 10 items, each answered using a 7-point Likert scale, where 1 means “Strongly disagree” and 7 means “Strongly agree”. The only exception is item 1, for which specific verbal labels are used: “No importance at all” for 1 and “Extremely important” for 7.

Additionally, the questionnaire begins with open-ended questions related to formal music studies, such as the age at which participants began their music education studies, the age at which they discontinued it (if applicable), the level achieved in their music studies (with options for elementary, intermediate, and advanced), and the instrument they play. A question was also added to identify the overall significance of music in a person’s life, asking them to respond: “For me, music is… (select the appropriate option)”, with response options including a high value, part of my identity, a profession, and a hobby. Participants were further asked if they consider themselves a “musician” (i.e., if it is part of their identity), with the option to answer YES/NO.

### 3.4. Structural Musical Profile (SMP)

The first element of the SMP describes the number of hours each musician currently devotes to music, as well as the hours they have devoted to it in the past, according to their personal history. It is based on the idea of measuring objective and quantifiable components of musical involvement. This dimension is based on time estimates and aims to be objective, as it asks for the number of hours spent on music per week, rather than an opinion about it. The questions in this dimension are divided into four time periods corresponding to academic and developmental stages (from childhood to age 12 years, from age 12 to 18 years, from age 18 years onward, and currently), asking about the number of hours per week spent on music (including regular lessons, autonomous study, and participation in various musical activities). This dimension allows for the description of the progression of musical involvement, detecting setbacks or even abandonment of music study, all measured by the number of hours dedicated to musical activities.

The second element of the SMP describes the different areas in which a musician may be involved, thus providing quantitative data on the number of different musical activities they engage in, and qualitative data on the specific musical aspects they are dedicated to. The items include the option to indicate participation the following activities: music study, playing an instrument, regularly attending a musical group (band, orchestra), music research, music creation, being part of an association to promote music, teaching music to others, and listening to music. These areas are assessed using a dichotomous response format (Yes/No). Additionally, there is an option to specify any activity not included in the aforementioned list.

### 3.5. Psychological Musical Profile (PMP)

The PMP scale describes cognitive–emotional aspects related to self-perception of the musical dimension. It consists of three factors or subscales. The first factor refers to self-perceptions regarding musical identity, specifically the self-perception of different elements that make up the musical self. While it was initially expected that the last four items would form a single factor related to reflection on the motivational elements and existential meaning of music, exploratory factor analysis (EFA) revealed a structure of two distinct factors. This structure suggests that the perception of music’s impact on life can be organized into two dimensions: one related to the feeling of burden or limitation, and the other related to the appreciation of its value and personal enrichment.

*Subscale: “Musical Self-Perception” (items 1–6)*

The items encompass the cognitive and emotional dimension of how an individual perceives themselves in relation to music: the perception of their own ability with music, the consideration of personal effort dedicated to it, the satisfaction derived from musical achievements, the importance of music in their life, the consideration of sacrifices made for music, and the gratitude felt for the opportunity to study music.

*Subscale: “Personal Cost of Music” (items 7–8)*

This factor captures the perception that the presence of music may imply certain negative aspects, referring to the sense of restriction or demands experienced by some individuals. Essentially, it evaluates the idea that, without music, life could be perceived as calmer or with greater freedom, reflecting a critical assessment of the personal commitment involved in its dedication.

*Subscale: “Existential Contribution of Music” (items 9–10)*

This factor measures the perception of music as a source of existential enrichment, captured through counterfactual items that encourage reflection on how life would be without it. Statements such as “life without music would be more boring” and “life without music would be emptier” invite participants to assess the role that music plays in providing meaning and satisfaction in their lives. By using this counterfactual approach, the items not only explore the importance of music in terms of immediate enjoyment but also highlight its fundamental role in counteracting feelings of emptiness and disengagement in daily life.

## 4. Discussion

The development of the MPQ has addressed the need for an instrument that allows for the brief and simple characterization of various aspects of musicians (both structural and psychological), with no similar questionnaires identified in the previous literature. The development of the questionnaire involved a research team of expert musician educators who, after reviewing the existing literature, crafted the items considering the full scope of the musical profile. Efforts were made to ensure that the questionnaire neither underrepresented nor overrepresented any particular dimension. The basic principles guiding the construction of any item bank were adhered to, ensuring that the items possessed representativeness, relevance, diversity, clarity, simplicity, and comprehensibility (Muñiz et al., 2005). Throughout the item development process, the expert in music psychology supervised the items to ensure optimal psychometric functioning.

Various statistical analyses were conducted on the PMP scale to ensure the development of an instrument that balances psychometric robustness with theoretical and practical evaluation needs. The Exploratory Factor Analysis (EFA) revealed a clear three-factor structure, which could be labeled according to their correspondence with conceptual elements of the psychological musical profile. One aspect to consider is that the items related to the existential meaning of music exhibited lower homogeneity indices—three of them showed moderate scores, while one presented a lower index (<0.2)—which may potentially affect the internal consistency of the scale. However, overall reliability remains sufficiently high and, therefore, the internal consistency of the entire scale is not compromised (Cronbach’s α = 0.782; McDonald’s ω = 0.788). Additionally, the counterfactual wording of these items may influence participant responses, which could account for the lower inter-item correlations. Therefore, it was decided to retain these items, as their removal would harm the content validity of the scale, particularly by excluding such a significant dimension as the existential meaning of music. Moreover, this counterfactual phrasing is not regarded as a limitation but rather as a potential strength, enabling the capture of nuanced interpretations in how individuals assign meaning to their musical experiences, thereby enriching the construct assessment and offering a more differentiated understanding of the psychological musical profile. These four items with lower homogeneity indices were organized into two subscales: *Personal Cost of Music* and *Existential Contribution of Music*. This structure suggests that the perceived impact of music on one’s life can be understood along two dimensions: one reflecting feelings of burden or limitation, and another emphasizing appreciation of music’s value and personal enrichment. Nevertheless, the final configuration of only two items per factor may represent a limitation for future Confirmatory Factor Analyses of the instrument. Future research could consider increasing the number of items in the factors to improve internal consistency and model validity. While it is possible to have factors with only two items, studies have shown that with three items per factor, confirmatory model estimates are more accurate and reliable (Avello-Martínez & Rodríguez-Monteagudo, 2020; Melia, 2008). Therefore, in case of low internal consistency, rather than eliminating items, it is suggested to add more items to strengthen the measurement of the construct and meet the methodological robustness requirements of CFA. This strategy would not only increase the reliability of the instrument but also avoid loss of relevant content.

On the other hand, it is considered that this questionnaire has not left any important aspects unrepresented, nor does it include items that are unrelated to the variable being measured, which would introduce irrelevant variance. Future theoretical reflections and subsequent confirmatory factor analyses will clarify whether the structure obtained for the PMP scale is maintained and whether additional content should be included to improve measurement.

Furthermore, the correlations obtained between the PMP scale, the level of music education attained, and the various areas of musical involvement indicate good criterion validity, as higher scores on the PMP scale are associated with achieving a higher level of music studies and greater dedication to music. Additionally, the correlations between the PMP scale and the General Musical Self-efficacy Scale (Ritchie & Williamon, 2007, 2011; Cuartero-Oliveros, 2019) have been consistent with expectations, showing significant relationships that suggest good convergent validity. This is because both scales assess related but distinct aspects. While our scale covers a broader range of content to characterize musicians, the General Musical Self-efficacy Scale focuses on a more specific construct.

## 5. Conclusions

In conclusion, this study has developed the MPQ, following in detail all the guidelines established by Muñiz and Fonseca-Pedrero (2019) for test creation. In light of the promising results obtained, it is considered that this questionnaire has the potential to be used in a wide range of research contexts within the fields of music psychology and music education. In this way, a potentially useful instrument for characterizing musicians is offered. Although there are scales and questionnaires that measure various aspects of the musical sphere, no instrument has been found that integrates different structural and psychological dimensions as the one we have developed does. The structural dimension has gathered basic elements of dedication in terms of time and musical facets. The psychological dimension has been constructed based on relevant theoretical assumptions in music education and the expertise of professionals in this field and music psychology, ensuring that it possesses appropriate psychometric properties. The instrument combines qualitative and quantitative elements, which provides broad coverage for the characterization of musicians.

Furthermore, the items have been created considering universal aspects of music, making it an instrument that can easily be extrapolated to musicians from other cultural and linguistic contexts. Furthermore, the MPQ is a brief questionnaire, compared to other similar questionnaires. This characteristic is considered useful, as they are much more versatile for both data collection and numerous statistical analyses that require greater brevity and consistency.

Therefore, this questionnaire not only provides a useful tool for professionals and researchers in the fields of music education and music psychology but it also opens the door to future research that could further enrich our understanding of the musical phenomenon from a psychological perspective. The next step in the advancement of the instrument at the research level would be validation through Confirmatory Factor Analysis (CFA), which would allow for the assessment of the internal structure of the scale and confirm its validity. It would be valuable to track the progress in the application of this questionnaire and the results derived from its use in different populations of musicians.

As for limitations of the present study, the underrepresentation of certain musical activities in the sample (e.g., music researchers) is noted, although such participants are less common than performers or music students. Therefore, future recruitment will continue in other contexts, such as university settings. It is recommended that future research expand the diversity of participants in terms of their musical areas to improve the characterization of musicians’ profiles, thereby achieving a better representation of the musical field.

### Practical Implications

The use of a structural and psychological musical profile questionnaire in the field of music education, especially in conservatories, has several practical implications that can benefit both students and teaching professionals. First, by assessing aspects such as the time dedicated to music, the diversity of musical activities, and students’ psychological perceptions, the questionnaire can help educators identify each student’s strengths and areas for improvement, enabling more personalized instruction tailored to their needs and potential.

Furthermore, by providing a more holistic view of the student’s structural and psychological musical profile, it becomes possible to identify aspects that impact their development, such as motivation, musical self-concept, or self-efficacy beliefs. This would enable educators to offer appropriate emotional and psychological support, both in guiding students and in developing strategies to enhance their student’s well-being and performance.

At the institutional level, the implementation of the questionnaire would allow conservatories and other educational institutions to obtain more accurate data on the characteristics and needs of their students, which could contribute to the improvement of educational programs and the creation of a more balanced and supportive learning environment. Additionally, it could be used to design continuous professional development programs for educators, enabling them to better understand the psychological and structural variables that influence musical learning and students’ mental health.

In conclusion, the MPQ presented in this study is an innovative and valuable tool for comprehensively assessing the various aspects that shape musicians’ engagement and well-being, offering new perspectives for both research in music psychology and practical application in educational and therapeutic settings.

## Figures and Tables

**Table 1:** Descriptive statistics items of the Psychological Music Profile Scale (PMP).

							If Item Dropped		
Item	Mean	SD	Skewness	Std. Error of Skewness	Kurtosis	Std. Error of Kurtosis	McDonald’s ω	Cronbach’s α	Item-Test Correlation
1	4.943	1.953	0.717	0.151	0.658	0.300	0.731	0.735	0.634
2	6.273	1.118	1.876	0.151	3.790	0.301	0.777	0.767	0.446
3	5.065	1.491	0.852	0.151	0.453	0.301	0.769	0.759	0.488
4	5.023	1.792	0.785	0.151	0.383	0.301	0.741	0.743	0.590
5	6.330	1.414	2.459	0.151	5.503	0.300	0.755	0.750	0.578
6	5.161	1.960	0.918	0.151	0.347	0.300	0.732	0.731	0.663
7	2.490	1.935	1.129	0.151	0.017	0.300	0.802	0.778	0.354
8	2.774	1.963	0.790	0.151	0.724	0.300	0.806	0.782	0.330
9	6.158	1.507	2.254	0.151	4.601	0.301	0.769	0.784	0.275
10	6.537	0.714	3.189	0.191	13.25	0.379	0.788	0.788	0.162

**Table 2:** Correlation matrix of the items and the total scale.

			1	2	3	4	5	6	7	8	9	10	Total Scale
Spearman’s rho	1	ρ Spearman	1.000										
		Sig.											
	2	ρ Spearman	0.550 **	1.000									
		Sig.	0.000										
	3	ρ Spearman	0.476 **	0.492 **	1.000								
		Sig.	0.000	0.000									
	4	ρ Spearman	0.461 **	0.371 **	0.411 **	1.000							
		Sig.	0.000	0.000	0.000								
	5	ρ Spearman	0.508 **	0.482 **	0.395 **	0.456 **	1.000						
		Sig.	0.000	0.000	0.000	0.000							
	6	ρ Spearman	0.512 **	0.281 **	0.243 **	0.413 **	0.389 **	1.000					
		Sig.	0.000	0.000	0.000	0.000	0.000						
	7	ρ Spearman	0.172 **	−0.070	0.028	0.167 **	0.011	0.420 **	1.000				
		Sig.	0.005	0.261	0.658	0.007	0.856	0.000					
	8	ρ Spearman	0.127 *	−0.049	0.013	0.153 *	0.057	0.442 **	0.699 **	1.000			
		Sig.	0.040	0.435	0.836	0.014	0.362	0.000	0.000				
	9	ρ Spearman	0.255 **	0.381 **	0.209 **	0.211 **	0.318 **	0.163 **	−0.016	−0.037	1.000		
		Sig.	0.000	0.000	0.001	0.001	0.000	0.009	0.794	0.553			
	10	ρ Spearman	0.169 **	0.278 **	0.167 **	0.160 **	0.211 **	0.087	−0.097	−0.112	0.503 **	1.000	
		Sig.	0.006	0.000	0.007	0.010	0.001	0.160	0.119	0.072	0.000		
	Total scale	ρ Spearman	0.698 **	0.497 **	0.499 **	0.624 **	0.545 **	0.749 **	0.557 **	0.547 **	0.371 **	0.230 **	1.000
		Sig.	0.000	0.000	0.000	0.000	0.000	0.000	0.000	0.000	0.000	0.000	

Note. * *p* < 0.05, ** *p* < 0.01.

**Table 3:** Factor loadings.

	Factor 1	Factor 2	Factor 3	Uniqueness
2	0.786			0.365
3	0.722			0.524
4	0.700			0.495
5	0.695			0.485
1	0.613			0.534
6	0.575			0.444
8		0.870		0.251
7		0.828		0.312
9			0.662	0.541
10			0.571	0.683

Note. Applied rotation method is oblimin.

## Data Availability

Data are available on request to the corresponding author.

## Appendix A

**Table A1:** Musical Profile Questionnaire (MPQ)-Spanish Version.

Instrucciones: A continuación, encontrarás una serie de preguntas para conocer mejor tu faceta de músico; responde con la mayor sinceridad posible e intenta no dejarte ninguna pregunta por responder.		
	Preguntas descriptivas músicos Questions	
1	Grado alcanzado en estudios reglados de música	Elemental/Medio/Superior
2	Instrumento	
3	¿A qué edad empezaste los estudios reglados de música?	Indicar edad en números
4	¿Sigues en la actualidad con estudios de música?	SI/NO
5	En caso de haber abandonado los estudios de música, indicar la edad en la que se abandonaron	Indicar edad en números
6	Ser músico forma parte de mi identidad (Me considero músico/a):	SI/NO
Perfil Musical Estructural (Tiempo) Structural Musical Profile (Time)—SMP		
1	Hasta los 12 años aproximadamente, el número de horas a la semana dedicado a la música (incluyendo clases ordinarias, dedicación de estudio autónomo y asistencia a actividades musicales varias) fue de… (nota: este dato es difícil de cuantificarse de modo exacto, por lo que se refiere a una estimación global)	Escribe el número de horas
2	Entre los 12 años y 18 aproximadamente, el número de horas a la semana dedicado a la música (incluyendo clases ordinarias, dedicación de estudio autónomo y asistencia a actividades musicales varias) fue de… (nota: este dato es difícil de cuantificarse de modo exacto, por lo que se refiere a una estimación global)	Escribe el número de horas
3	A partir de los 18 años aproximadamente, el número de horas a la semana dedicado a la música (incluyendo clases ordinarias, dedicación de estudio autónomo y asistencia a actividades musicales varias) fue de…(nota: este dato es difícil de cuantificarse de modo exacto, por lo que se refiere a una estimación global)	Escribe el número de horas
4	En la actualidad mi dedicación de tiempo a la música es (indica aproximadamente las horas a la semana): …(nota: este dato es difícil de cuantificarse de modo exacto, por lo que se refiere a una estimación global)	Escribe el número de horas
	Perfil Musical Estructural (Ámbitos) Structural Musical Profile (Areas)—SMP	
1	En la actualidad, dedico parte de mi tiempo a estudiar música	SI/NO
2	En la actualidad, dedico parte de mi tiempo a tocar un instrumento	SI/NO
3	En la actualidad asisto con regularidad a alguna agrupación musical (banda, orquesta…)	SI/NO
4	Me dedico a la investigación en música	SI/NO
5	Realizo creaciones musicales	SI/NO
6	Formo parte de alguna asociación cultural dedicada a promover la música	SI/NO
7	Escucho música frecuentemente	SI/NO
8	Enseño música a otros	SI/NO
9	Indica si realizas alguna otra actividad en relación a la música no mencionada anteriormente	Pregunta abierta
	Escala Perfil Musical Psicológico (PMP) Psychological Musical Profile Scale (PMP)	
	Subescala “Autopercepción musical” Subscale: “Musical Self-Perception”	
1	La importancia de la música en mi vida es	Absolutamente ninguna importancia (1)–Extremadamente importante (7)
2	Dedico un gran esfuerzo en actividades relacionadas con la música	Nada de acuerdo (1)–Totalmente de acuerdo (7)
3	Considero que tengo una gran capacidad para la música	Nada de acuerdo (1)–Totalmente de acuerdo (7)
4	Estoy satisfecho con los logros que he alcanzado en relación con la música	Nada de acuerdo (1)–Totalmente de acuerdo (7)
5	Estoy agradecido por haber tenido la oportunidad de estudiar música	Nada de acuerdo (1)–Totalmente de acuerdo (7)
6	Considero que la música ha requerido mucho sacrificio en mi vida	Nada de acuerdo (1)–Totalmente de acuerdo (7)
	Subescala “Coste personal de la música” Subscale: “Personal Cost of Music”	
7	En ocasiones me he planteado que la vida sin la música sería más tranquila	Nada de acuerdo (1)–Totalmente de acuerdo (7)
8	En ocasiones me he planteado que la vida sin la música hubiese gozado de más libertad	Nada de acuerdo (1)–Totalmente de acuerdo (7)
	Subescala “Aportación existencial de la música” Subscale: “Existential Contribution of Music”	
9	En ocasiones me he planteado que la vida sin la música sería más vacía	Nada de acuerdo (1)–Totalmente de acuerdo (7)
10	En ocasiones me he planteado que la vida sin la música sería más aburrida	Nada de acuerdo (1)–Totalmente de acuerdo (7)
